# Effects of *Piriformospora indica* and arbuscular mycorrhizal fungus on growth and physiology of *Moringa oleifera* under low-temperature stress

**DOI:** 10.1515/biol-2025-1111

**Published:** 2025-06-13

**Authors:** Guo Zhou, Sini Wu, Meichun Qiu, Yingtong Long, Qian He, Junjie Zhang

**Affiliations:** College of Forestry and Landscape Architecture, South China Agricultural University, Guangzhou, 510642, China; Guangdong Key Laboratory for Innovative Development and Utilization of Forest Plant Germplasm, Guangzhou, 510642, China; Guangdong Province Research Center of Woody Forage Engineering Technology, Guangzhou, 510642, China

**Keywords:** rhizosphere growth-promoting microbe, *Moringa*, physiological characteristics, cold resistance

## Abstract

*Moringa* is a perennial tree with high nutritional value, and it is drought tolerant and barren but has poor cold resistance during the seedling stage. This study selected *Piriformospora indica* (PI) and arbuscular mycorrhizal fungus (AM) as inoculants, *Moringa* seedlings were inoculated, and their growth and physiological responses were evaluated under different low-temperature stress times. The research results show that PI and AM can symbiotically associate with *Moringa* successfully and promote their nutritional growth. At low temperature, Moringa inoculated with PI and AM exhibited better physiological resistance. However, the effect of mixed inoculation of PI and AM is not as significant as that of single inoculation of any strain. Inoculating plant growth-promoting rhizobia (PGPR) reduced the richness of fungal communities and the number of unique operational taxonomic units (OTUs), with PI being the most prominent. Vaccination with PGPR also increased bacterial diversity, richness, and the number of unique OTUs, with AM inoculation showing the most prominent performance. This suggests that *Moringa* seedling growth and responsiveness to low-temperature stress are significantly influenced by PGPR, and there may be interactions between different bacterial strains. The results suggest that PGPR can improve the yield and quality of *Moringa* by promoting growth and regulating stress resistance.

## 1 Introduction

*Moringa oleifera* Lam. is a perennial tree of the *Moringa* genus in the Moringaceae family, native to India [1]. Due to its rapid growth, strong adaptability, and high nutritional value, it is widely planted in tropical and subtropical regions [2]. Due to its unique nutritional value and wide range of uses, *Moringa* is praised by Western scientists as the “magical tree” [3]. Therefore, it has been found that *Moringa* leaves can be developed into new feed resources and applied to the livestock and poultry breeding industry [4,5,6]. However, *Moringa* has poor cold resistance, and severe cold may cause the aboveground parts of *Moringa* to die, which significantly limits its promotion and application.

Inoculating plant growth-promoting rhizobia (PGPR) usually refers to bacteria or fungi that can colonize the rhizosphere of plants and promote the growth of plants. PGPR encompasses not only rhizobia but also various other types of beneficial bacteria and fungi that colonize the rhizosphere and enhance plant growth, including *Piriformospora indic*a (PI) and arbuscular mycorrhizal fungus (AM). PGPR has the most significant influence in the agricultural field, and the application of the PGPR mechanism can reduce the amount of nitrogen fertilizer, reduce production costs, and improve crop yield and quality. Secondly, in horticulture and forestry, the PGPR mechanism can promote root development and improve the survival rate of transplanting, which can be used for the cultivation of flowers and seedlings.

PI is an endophytic fungus belonging to the Sebacinales order of the Basidiomycota phylum [7]. Research has shown that the PI can interact symbiotically with various crops such as rice and wheat, promoting the increase of plant biomass and resistance stability in environments such as drought stress, salt stress, and heavy metal stress [8,9]. Arbuscular mycorrhizal fungi (AMF) are a kind of fungi that have a symbiotic relationship with plant roots, and Funnelifor mismosseae in AMF. Originally known as Glomus mosseae, which belongs to the genus Glomus in Glomeraceae, it is an AMF in soil. Funneliformes mossae (FM), formerly known as *Fusarium graminearum*, is a common fungus in soil with strong environmental adaptability. It is easy to infect plant roots and has a good symbiotic relationship with most plants [10,11]. Research has shown that arbuscular mycorrhizal fungi can enhance the nutrient absorption capacity of plants and increase antioxidant enzyme activity, thereby protecting membrane integrity and stabilizing protein resistance to stress [12].

PI and AM, as beneficial bacteria and fungi [13], have been increasingly studied, but there is relatively little research on the effects of inoculation of *Moringa* and beneficial fungi and bacteria on the growth of *Moringa*. At present, there is no article that simultaneously inoculates PI and AM on *M. oleifera*. Some studies have found that the colonization of AM has not changed the total growth of *M. oleifera*, which is in sharp contrast with previous studies that showed positive effects [14]. But the article also found that: AM inoculation improved the growth and biomass of two *Moringa* species [15]. PI confers banana with enhanced cold resistance by cumulative antioxidant capacity, ss accumulation, And the expression of cold-responsive genes in leaves [16]. In addition, it is found that inoculating fungi can improve the survival rate of wheat in low-temperature environment [17]. This enriches the research content of the interaction mechanism between plants and beneficial fungi and bacteria, provides a new case for deeply understanding how to establish and operate the symbiotic system, provides new ways and ideas for improving the study of plant stress resistance, and promotes the expansion and application of this field in other plant varieties.

*M. oleifera* seedlings showed poor cold resistance at low temperature, which reduced its survival rate. Under low temperature stress, the cell membrane integrity of *M. oleifera* was destroyed, the antioxidant defense system was unbalanced, and the photosynthetic activity was inhibited. These findings clearly highlight the problem gap in *M. oleifera* cultivation and emphasize the urgent need for effective strategies to enhance its cold resistance. PI and AM fungi can form a symbiotic relationship with plant roots, which can enhance the production of antioxidants in plants and help improve the ability of plants to resist cold stress. So this study conducted PI and AM inoculation with *Moringa*. By analyzing the effects of inoculation on *Moringa* seedlings, a theoretical basis was provided for fully utilizing microbial resources to promote plant growth and improve plant stress resistance. By applying this inoculation method, we aim to significantly improve *Moringa*’s cold resistance during seedling cultivation and afforestation, thereby overcoming the limitations posed by its inherent cold sensitivity.

16S amplicon sequencing refers to the amplification of 16S of microorganisms in the environment using suitable universal primers 16SrDNA/18SrDNA/ITS high-variable regions or functional genes, using high-throughput sequencing technology to detect the sequence variation and abundance information of PCR products, analyze the diversity and distribution of microbial communities in this environment, so as to reveal the types, relative abundance, and evolutionary relationships of microorganisms in environmental samples.

## 2 Materials and methods

### 2.1 Plant material and treatments

#### 2.1.1 Test materials and processing

Soak *Moringa* seeds in a solution containing carbendazim with a concentration of 0.1% for 4 h to kill fungus, and then place them in a suitable temperature incubator for germination [18]. After 3–4 days, remove and sow the seeds, select healthy *Moringa* seedlings that grow consistently, and transplant them into potted plants. In addition to the control, the seedlings were inoculated with three microbial treatments, PI, AM, and AP, a combination of both (AM + PI) [19].

PI comes from the Forest Genetics and Breeding Laboratory of the College of Forestry and Landscape Architecture, South China Agricultural University. The inoculant is a 5 mm bacterial cake cultured on PDA solid medium for 7 days. AM was purchased from the Guangdong Institute of Microbiology, and the experimental inoculum consisted of a mixture of spores(70 spores/g soil), infected root segments, propagation substrate, and mycelium.

### 2.2 Experimental design

In this experiment, the peat soil pot planting method was adopted, and the soil was put into a sterilizer at 120° for high-temperature sterilization for 30 min, with 1 plant per pot. There are four treatments, namely control, inoculation with Pi, inoculation with AM, and simultaneous inoculation with PI and AM. Each treatment involves five seedlings, repeated three times [20]. The PI treatment group was inoculated with six pieces of 5 mm PI per pot, the AM treatment group was inoculated with 10 g of AM agent per pot, the AP treatment group was inoculated with three pieces of 5 mm PI and 5 g of AM, and the CK treatment group was not inoculated with fungal and bacteria. Low temperature stress was carried out on the 85th day after inoculation with PI and AM. Low-temperature stressed seedlings were placed in the sterilized artificial climate incubator and subjected to gradient cooling exercises at 20, 15, and 10°C for 4 days each. Afterward, they were cooled down at a rate of 2°C/h to 4°C and kept constant [21].

### 2.3 Characteristic determination method

Ninety days after inoculation with PI and AM, 50 fresh root segments of approximately 1 cm were selected for each treatment, and the mycorrhizal infection rate was determined using the triple blue staining method. Using vernier calipers, tape measures, etc., to measure plant height, ground diameter, and leaf number (excluding cotyledons), and using Li-6400XT photosynthetic fluorescence analyzer to measure the photosynthesis of the third to fifth functional leaves of *Moringa* from top to bottom; after separating the roots, stems, and leaves of the plant, use a root scanner to scan the roots and leaves (excluding cotyledons), and analyze them using WINRHIZO root analysis software to obtain their root surface area and leaf surface area; Subsequently, it was placed in a 100°C oven for high-temperature withering for 30 min and then placed in a 50°C oven for constant temperature drying to a constant weight. Then, an electronic balance was used to weigh the roots, stems, and leaves (excluding cotyledons) to obtain biomass. In the low-temperature stress treatment, six pots of *Moringa* with consistent growth were selected for each treatment, and 1 g of mature leaves were taken for physiological index measurement. Physiological indicator materials used for measuring the content of various enzymes and chlorophyll are stored at −80°C ultra-low temperature for future use [22]. The kits for testing plants PRO, MDA, H_2_O_2_, superoxide dismutase (SOD), peroxidase (POD), and catalase (CAT) all came from China Suzhou Keming Biotechnology Co., Ltd.

### 2.4 Soil microbial community detection

After 90 days of inoculation, soil samples were collected at a depth of 5 cm around the ground diameter of potted *Moringa* plants using a three-point sampling method. Three pots were collected for each treatment (nonmixed sampling). Place the collected soil samples in sterile self-sealing bags and store them in a −80°C ultra-low temperature freezer for microbial diversity testing. The collected soil samples will be sent to Guangzhou Gidio Co., Ltd., for determination using lllumina Miseq™ sequencing technology. The 16S rRNA gene of bacteria and the ITS gene of fungi will be amplified and sequenced.

### 2.5 Data statistics and analysis

Data acquired in the experiments were sorted using Microsoft Office Excel (Microsoft Crop., Redmond, WA, USA), and then, statistical analyses were performed with SPSS software (Version 26.0; SPSS Inc., Chicago, IL, USA). *P* values ≤ 0.05 were considered statistically significant.

## 3 Results

### 3.1 PI and AM form symbiotic associations with roots in *Moringa*

#### 3.1.1 Root colonization by PI and AM in *Moringa*

**Figure 1:**
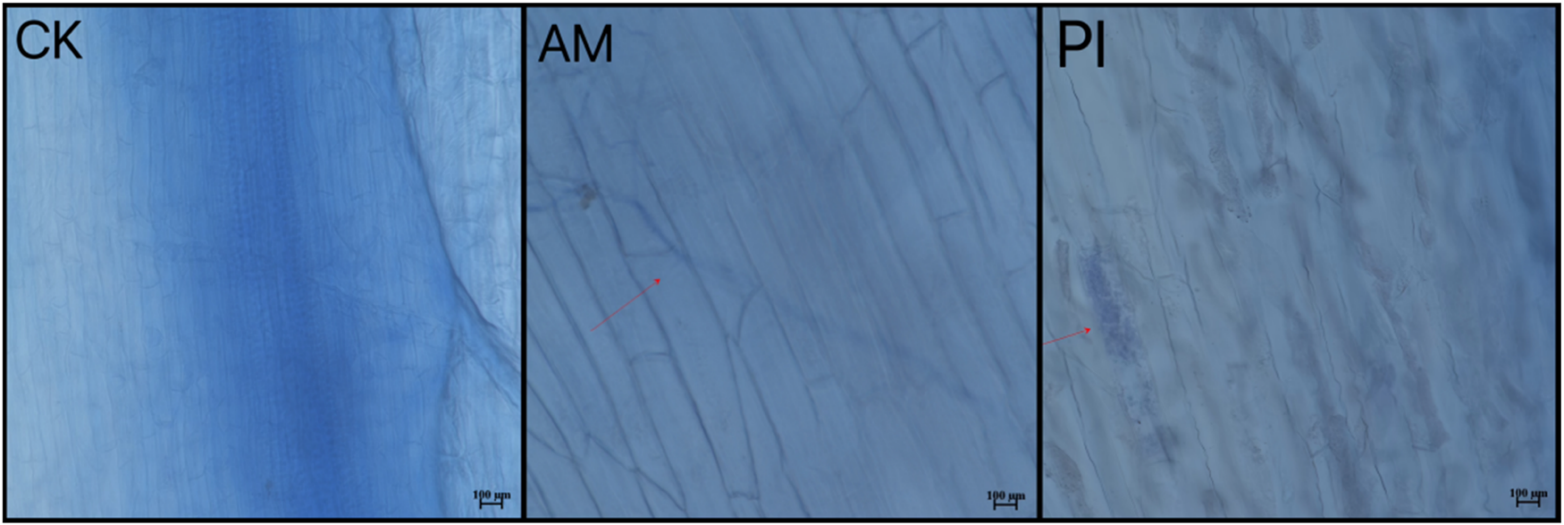
Observation under a microscope of the CK group without inoculation of *Moringa*, inoculation with AM and PI bacteria.

After 90 days of symbiosis between PI and *Moringa* root system, bead-like hyphae were observed under a 40× biological microscope, with elliptical thick-walled spores clustered together, as indicated by the arrows in Figure 1. After 90 days of inoculation with AM, Y-shaped clustered hyphae can be observed, as indicated by the arrows in Figure 1. The combination of AP and beneficial fungi and bacteria with *Moringa* includes two separate inoculation methods, mainly based on hyphal structure, followed by pear-shaped spores. This indicates that PI and AM can form a good mycorrhizal symbiotic relationship with *Moringa*.

### 3.2 The effect of inoculating AM and PI on the growth morphology of *Moringa*

#### 3.2.1 Effect on growth index

**Figure 2:**
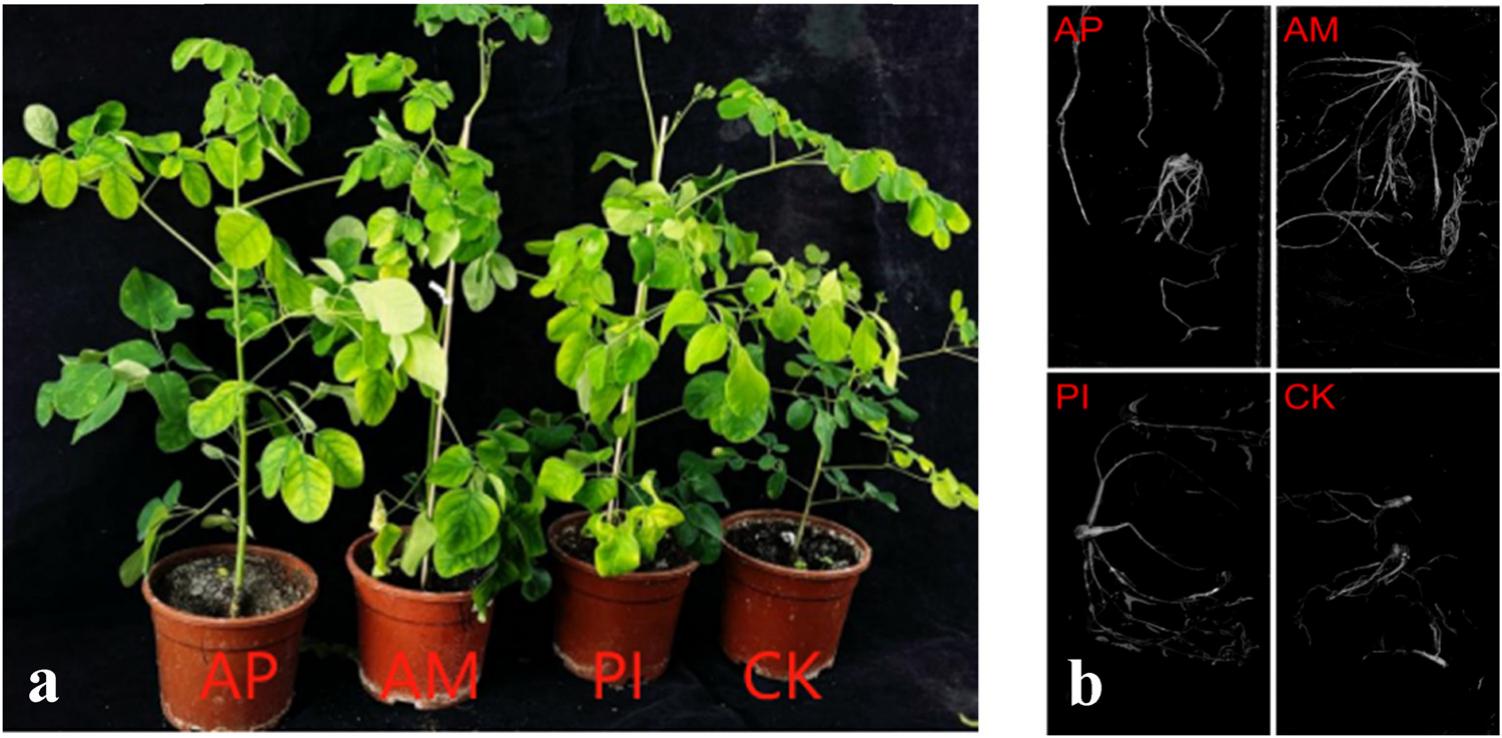
60 days after inoculation with PI and AM (a). Root surface area of various treatments of *M. oleifera* (b).

Plant height, ground diameter, and number of leaves are important indicators reflecting the level of plant growth(see Figure 2). Take 15 plants from each treatment in this experiment, analyze the average value, and explore the effects of different beneficial fungi and bacteria on the growth of *Moringa* (Table 1). In summary, PI treatment accelerated plant height growth, while AM treatment accelerated ground diameter growth and increased the number of leaves.

**Table 1:** Effects of inoculation with endophytic fungi on the infection rate and growth indicators of *Moringa* Mycorrhiza

Index	Treatments*			
	CK	PI	AM	AP
Colonization level (%)	0	35.03 ± 0.73a	40.08 ± 8.45a	14.54 ± 4.05b
Plant height (cm)	24.78 ± 1.94c	31.47 ± 2.44a	31.10 ± 2.85a	28.20 ± 3.31b
Ground diameter (mm)	3.03 ± 0.43c	3.32 ± 0.23ab	4.15 ± 0.42a	2.68 ± 0.22c
Number of leaves/plant (piece)	8.4 ± 0.61bc	9.7 ± 0.66ab	10.8 ± 0.92a	6.5 ± 0.65c
Surface area (cm^2^)	103.50 ± 3.17c	144.50 ± 3.75b	180.50 ± 2.02a	109.63 ± 5.87c
Root surface area (cm^2^)	167.73 ± 16.56c	281.48 ± 4.54b	346.76 ± 3.68a	202.55 ± 22.83c
Mean root diameter (cm)	0.0828 ± 0.0036c	0.1204 ± 0.0003b	0.1400 ± 0.0023a	0.0996 ± 0.0054d
Total root length (cm)	96.43 ± 3.146c	133.81 ± 513.01b	186.25 ± 2.72a	105.38 ± 4.07c
Total biomass (g)	1.43 ± 0.06c	1.67 ± 0.04ab	2.14 ± 0.05a	1.5 ± 0.07c
Root–shoot ratio (%)	0.09 ± 0.01b	0.10 ± 0.01ab	0.17 ± 0.012a	0.12 ± 0.012ab

*Means followed by the same letter in the same column are not significantly different from each other at *P* ≤ 0.05 level, according to Duncan’s multiple range test. The same goes for the other tables. abcd stands for significance and is usually used to indicate whether the differences between different groups are significant.

In terms of height, the impact on plant height was most significant in the PI treatment group, with an increase of 26.99% compared to CK, followed by AM, AP, and CK;

In terms of ground diameter, the order of influence on ground diameter is AM > PI > CK > AP.

In terms of the number of plant leaves, the AM treatment group significantly increased the leaf number of *Moringa* by 28.57% compared to the CK group in terms of its impact on leaf number.

In terms of the surface area of roots and leaves, the AM treatment raised the surface area of the leaves by 74.39% and the root surface area by 106% when compared to CK.

In terms of the average root diameter, except for the AM group, the PI and AP group had no significant effect on the average root diameter. The AM group’s average root diameter was 75% greater than the non-inoculated group’s;

In terms of the total root length, endophytic bacterial inoculation significantly increases the overall length of the roots. Compared to the infected treatment, the non-inoculated treatment’s total root length was noticeably shorter. The AM, PI, and AP treatments increased by 92.88, 39.28, and 9.28%, respectively, compared to the control treatment.

In terms of the root–shoot ratio, the root–shoot ratio of *Moringa* is obtained by drying them in a drying oven to a constant weight. The root–shoot ratio of each treatment ranged from 0.09 to 0.17, and the root–shoot ratio of the inoculant treatment and the not-inoculated treatment differed significantly. Among them, the root–shoot ratio of the AM-inoculated treatment group was higher than that of the non-inoculated treatment group.

This indicates that inoculation with endophytic bacteria increased the biomass of *Moringa* to varying degrees and allocated more biomass to the aboveground parts of *Moringa*.

#### 3.2.2 Effect on photosynthetic index

After 60 days of inoculation with endophytic bacteria, the determination results of photosynthetic parameters in comparison with non-inoculated *Moringa* plants are shown in Table 2. There is a significant difference compared with the control treatment, and the net photosynthetic rates of PI, AM, and AP treatments increased by 5, 65, and 45%, respectively; there was no significant difference in transpiration rate between the control group and the CK group, but the transpiration rate increased significantly after inoculation with PI bacteria; after inoculation with PI or AM, the intercellular CO_2_ of *Moringa* trees showed an increase of 2.2 and 5.0%, respectively, while the treatment of simultaneous inoculation with both decreased by 7.9%; the stomatal conductance of *Moringa* trees inoculated with PI or AM showed a significant decrease, with reductions of 41.2 and 31.4% compared to the CK group, respectively. However, the treatment in the AP group did not show a significant decrease, with a reduction of 9.8%. Endophytic bacteria have an impact on the photosynthetic parameters of *Moringa* leaves.

**Table 2:** Effects of endophytic bacteria inoculation on light response parameters of *Moringa*

Index	Treatments*			
	CK	PI	AM	AP
Net photosynthetic rate (μmol m^−2^ s^−1^)	0.20 ± 0.03b	0.21 ± 0.02b	0.33 ± 0.01a	0.29 ± 0.02c
Transpiration rate (mmol m^−2^ s^−1^)	0.11 ± 0.02a	0.26 ± 0.18a	0.080 ± 0.01a	0.10 ± 0.01a
Intercellular CO_2_ concentration (μL L^−1^)	426.533 ± 16.00a	435.96 ± 17.49a	447.66 ± 19.91a	392.87 ± 15.82a
Stomatal conductance (mol H_2_O m^−2^ s^−1^)	0.0051 ± 0.0009a	0.0030 ± 0.0003a	0.0035 ± 0.0004a	0.0046 ± 0.0004a

*Means followed by the same letter in the same column are not significantly different from each other at *P* ≤ 0.05 level, according to Duncan’s multiple range test. The same goes for the other tables. abcd stands for significance and is usually used to indicate whether the differences between different groups are significant.

### 3.3 Effects of AM and PI inoculation on physiological resistance of *Moringa* under cold stress

**Figure 3:**
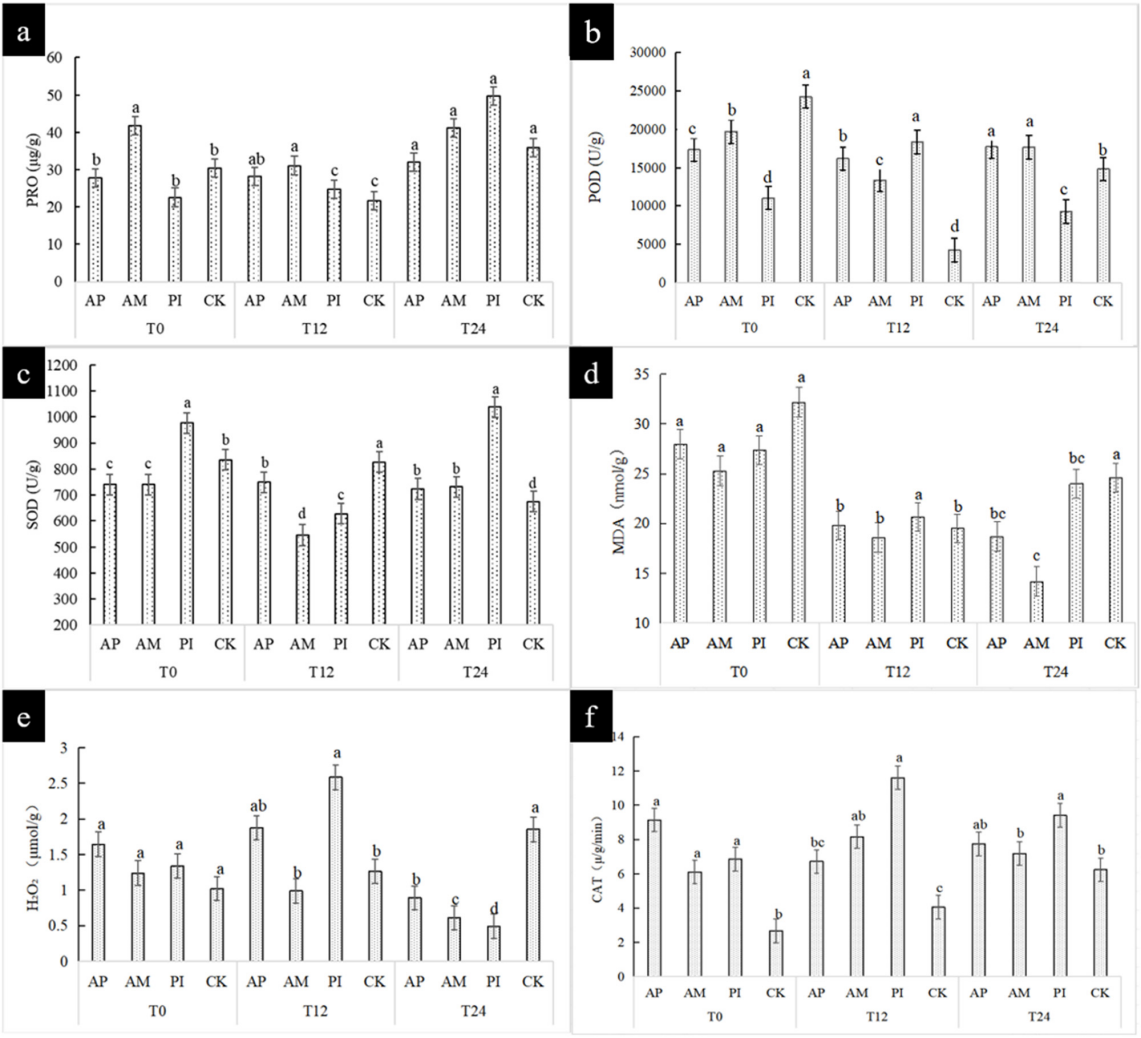
Plant physiological index (a)–(f) reflects the determination of physiological and biochemical parameters of *Moringa oleifera* injury.

All four treatments showed cold stress symptoms and the leaves began to wilt and dewater after being treated at 4°C for 12 h. The degree of cold damage increased with the extension of treatment time. However, the symptoms after vaccination were significantly better than those of the control group. Figure 3 shows the changes in the content of physiological indexes of *Moringa* inoculated with different strains at low temperatures (4°C) for 0 h (T0), 12 h (T12), and 24 h (T24), respectively.

With the extension of treatment time, the degree of freezing damage increased. However, the vaccinated symptoms were significantly better than those in the control group. With the increase in freezing time, the proline content of all treatment groups showed an increasing trend, and the PI treatment group was the most significant, which increased by 39% compared with CK. At room temperature, there was no significant difference in the concentration of hydrogen peroxide in seedlings. Under low-temperature stress, the concentration of hydrogen peroxide first increased and then decreased. With the extension of low-temperature treatment time, the difference between treatments became more and more significant. After 24 h of low-temperature stress, the concentration of hydrogen peroxide in plants inoculated with Pi was the lowest, which was significantly different from the other three treatments, the PI content was 74% less than CK, indicating that PI had a great influence on the hydrogen peroxide content. At room temperature, the MDA content in the control group was significantly 20% higher than that in the inoculation group. After 12 h of treatment, all treatment groups tended to be stable. After 24 h of treatment, the MDA content in plants inoculated with AM was the lowest, which was 42% less than CK, indicating that AM could greatly reduce the MDA content. Inoculation alleviated the damage of the leaf cell membrane of *Moringa* at low temperatures. This may be due to the activation of the antioxidant defense system in plants after inoculation with AM fungi. Antioxidants can remove excessive ROS in time, thus reducing MDA content and alleviating the damage of low temperatures to the cell membrane of *M. oleifera* leaves. After 24 h of low-temperature stress, the contents of CAT and SOD tended to be stable, and the contents of CAT and SOD in the treatment group were significantly higher than those in the control group by 30 and 23%. After 24 h of low temperature, the content of POD in the inoculation group increased by 19–22% except the PI group. The activity of SOD, POD, and CAT was increased by inoculation, which enhanced the plant’s ability to withstand adversity. For the increase of POD content in the inoculation group except the PI group, it may be because different inoculation treatments have different activation modes and degrees of plant antioxidant system. However, the PI group may not cause an obvious increase in POD content because of its interaction with plants, or its regulation and control focus on plant antioxidant system is more on other aspects, such as giving priority to enhancing the activities of CAT and SOD.

### 3.4 Effects of AM and PI inoculation on soil microbial community of *Moringa*

**Figure 4:**
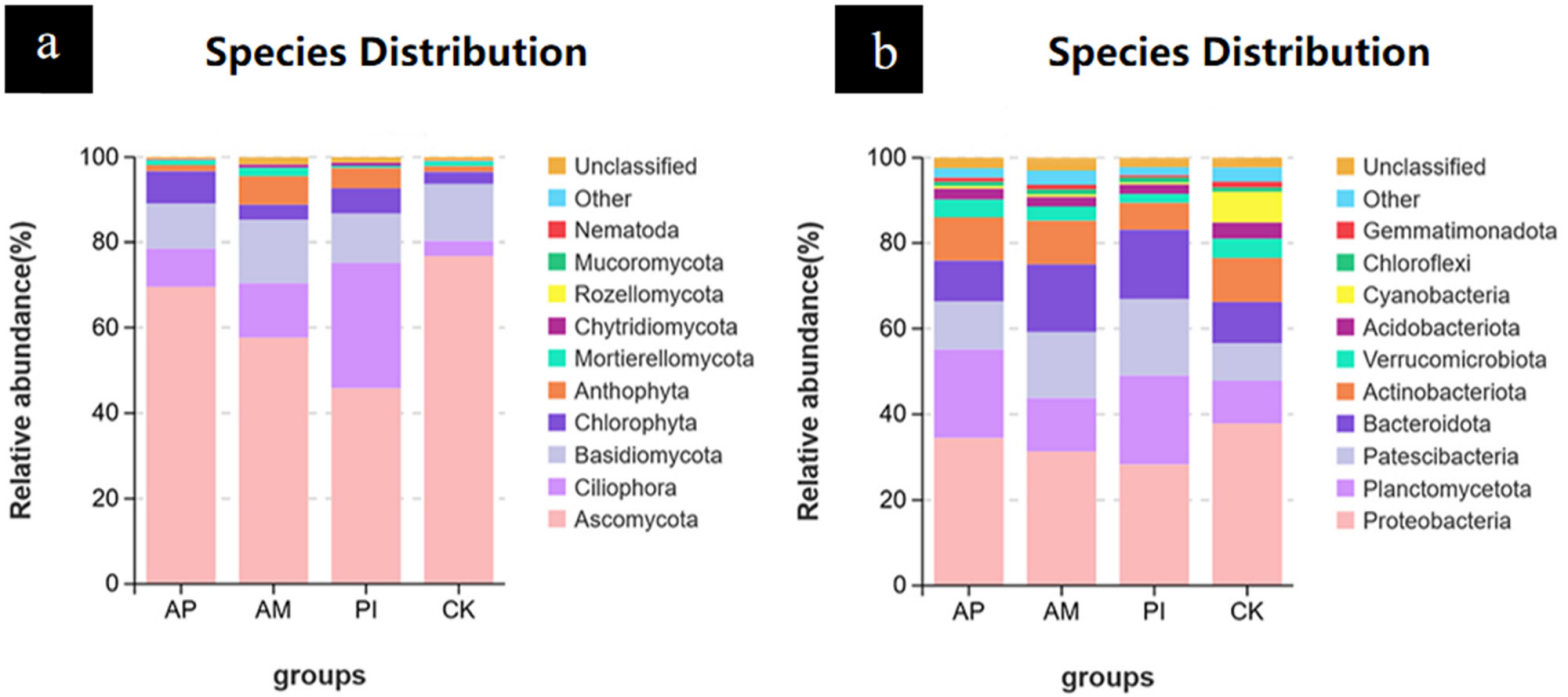
Composition of soil fungal (a) and bacterial (b) communities in different treatments of *Moringa* at gate level.

As can be seen from Figure 4a, the dominant fungal phyla of the four treatments were consistent in the composition of major species, but there were some differences in relative abundance. The three treatment groups infected with bacteria enriched fungal species to varying degrees, among which PI was the most significant. The top ten bacteria at the gate level were detected. As shown in Figure 4b, compared with CK, the relative abundance of Proteobacteria decreased in the inoculation group, while that of pontomyces, patella, and Bacteroidetes increased. Except for Proteobacteria, the relative abundance of other inoculation groups increased compared with the control group.

Indicating that bacteria-inoculation decreased the richness and evenness of fungal communities in soil, the average values of Sobs, ACE, and Chao1 indices in the control treatment were all greater than those in the bacteria-inoculation treatment. The Shannon and Simpson indices of AP, AM, and PI treatments were higher than those of the control group, indicating that inoculation of processed groups could improve the diversity of soil fungal community. The bacterial richness and diversity in the AM and AP groups were higher than those in the control group, as evidenced by the higher average values of the Sobs, ACE, Chao1, Shannon, and Simpson index of the bacterial communities in those groups. The Shannon index of the AM treatment group was the highest, indicating that AM inoculation was the most prominent in improving soil bacterial diversity.

**Figure 5:**
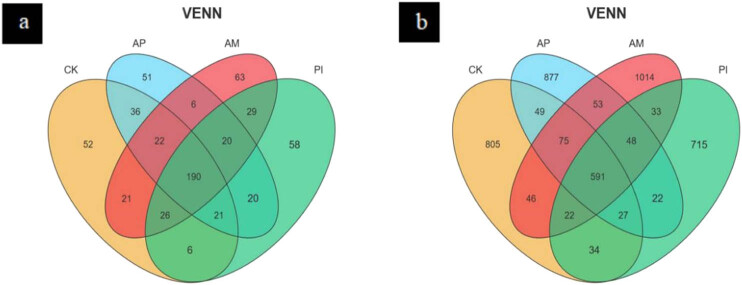
OTUs are common and endemic to different treated soil fungi (a) and bacteria (b).

Species Venn diagrams can be used to visualize the similarity and overlap of OTU numbers and composition of species. In a Venn diagram, each color represents a different process, and the number of OTUs shared by the groupings is shown by the numbers in the overlapping segments. According to Figure 5a, the number of OTUs of soil fungi under different treatments showed that the total number of OTUs of soil fungi was 621, and the total number of OTUs of the four treatments was 238. Among them, the unique OTUs of CK, AP, AM, and PI accounted for 8.37, 8.21, 10.14, and 9.33% of the total OTUS. It can be seen that AM is the highest, and CK and AP are the least significant, respectively. The OTU number of soil bacteria under different treatments was analyzed, as shown in Figure 5b. The total OTU number of soil bacteria was 4,411, and the total number of bacterial OTUs in the four treatments was 767, among which the OTUs specific to CK, AP, AM, and PI accounted for 18.25, 19.89, 22.99, and 16.2%, respectively. It can be seen that AM is the highest, and CK and PI are the least significant. Inoculation of PI and AM could not significantly increase the OUT number of soil fungal community and might even inhibit it but could increase the OTUs number of soil bacterial community. Overall, inoculation raised the diversity and richness of the bacterial population in the soil to varied degrees while decreasing the diversity and richness of the fungus community.

## 4 Discussion

In general, the higher the concentration of proline, the higher the concentration of soluble glucose, and proline can maintain the balance activity of intracellular enzymes, protect cells, accelerate plant energy recovery, and thus improve plant cold tolerance [23,24]. The results of this study showed that with the extension of low-temperature stress time, the proline content of *Moringa* in the inoculation group of piriformis indica and the double bacteria inoculation group showed a gradual increase trend, while it decreased first and then increased in the control group. This indicated that *Moringa* could achieve resistance stability by increasing proline content under low-temperature stress for a long time. It is worth mentioning that the inoculated pyriformis group was 120.41% higher than the initial value after 24 h of low-temperature stress, and the content of proline was significantly increased.

When PI or AM are inoculated alone, the absorption of phosphorus can be increased, which is essential for the synthesis of ATP and NADPH, which are the key molecules in the light-dependent reaction of photosynthesis, but the carbon sequestration rate in the Calvin cycle may not keep up immediately. Therefore, the consumption of CO_2_ in cells decreases, leading to an increase in the level of CO_2_ between cells; When PI and AM are inoculated at the same time, the combined effect of them may improve the metabolic efficiency of plants. Optimize the supply of various nutrients, enhance the activity of key photosynthetic enzymes (such as Rubisco), and improve the overall efficiency of the photosynthetic electron transfer chain. This will lead to faster consumption of CO_2_ in the process of carbon fixation in the Calvin cycle, resulting in the decrease in intercellular CO_2_ concentration [25].

Antioxidant enzymes are SOD, POD, CAT, polyphenol oxidase, phenylalanine aminolyase, thioredoxin POD, glutathione POD, and other general names [26]. Antioxidant enzymes can convert excessive reactive oxygen species (ROS) and free radicals (i.e., free radicals) in the body into less toxic or harmless substances, balancing the active oxygen content in the body, and its value can indicate that the plant has been stressed by the external environment [27,28,29]. The results of this study showed that with the extension of low-temperature stress time, the contents of POD, SOD, and CAT of the antibacterial treatment group were higher than those of the CK control group. These results indicated that inoculated PI and AM could resist the inhibitory effect of low-temperature stress on growth by increasing the activity of antioxidant enzymes to a certain extent; however, the control system of antioxidant enzymes in plants was harmed and their activity decreased as the duration of low temperatures increased.

Under cold stress, the permeability of cell membrane will increase, polyunsaturated fatty acids will fall off, and then, membrane lipid peroxy will be formed on the cell membrane, and the content of malondialdehyde will increase [30]. This study showed that with the extension of low-temperature stress time, the contents of MDA and H_2_O_2_ in potted *Moringa* showed a decreasing trend, and the contents of the inoculation group were lower than that of the control group under various stress duration periods, which may be due to the increased SOD, POD, and CAT activities of the inoculated PI and AM. As a result, the scavenging ability of ROS in plant cells is improved, which balances the production and scavenging of free radicals in cells, and reduces the peroxidation of membrane lipids, alleviating the cell membrane damage caused by low-temperature [31]. Low-temperature stress increased the peroxidation degree of Moringala moringala’s cell membrane, but the treated seedlings could effectively reduce the contents of MDA and H_2_O_2_ in leaves, thereby alleviating the damage of stress on the permeability of the lipid membrane of plant cells and improving the stress resistance of plants [32].

The inoculation of PI and AM decreased the richness of the fungal community and the number of unique OTUs but increased the diversity. The inoculation of PI and AM increased the abundance of ciliata, basidiomycetes, chlorophyta, and xanthophyta, and decreased the abundance of ascomycetes, with PI being the most prominent. Inoculation with PI and AM increased the diversity and abundance of bacteria and the number of unique OTUs, and the preponderant Bacteroides, patella, and Bacteroidetes had the highest abundance. It promoted the metabolism of soil bacteria, such as terpenoids and polyketones, biosynthesis of other secondary metabolites, and cell activity, among which inoculation with AM was the most prominent.

## 5 Conclusion

In terms of physiological characteristics, The treatment of PI under cold stress is better than the normal content and other treatment groups, indicating that inoculation of PI in a cold environment is a potential ideal technology to improve the growth and development of *Moringa*. PI treatment is slightly better than AM treatment, which may be because PI can improve the stress resistance of plants and resist stress by stimulating the activity of antioxidant enzymes and the accumulation of osmotic adjustment substances.

In terms of growth and development, inoculation of AM alone has a more positive effect on *Moringa* growth, which may be due to AM’s extensive extraroot mycelium network, which can make the host plants get more soil nutrients and water.

Compared with the current research, the study on the effect of combined inoculation of bacteria and fungi on *Moringa* growth also involves cold-resistant physiology and soil microbial community. The relationship between beneficial fungi and bacteria and *Moringa* was explored from multiple dimensions. In the study of cold resistance physiology, the changes in cell membrane permeability and antioxidant enzyme activity at low temperatures are involved, and the study of soil microbial community can reveal the influence of beneficial fungi and bacteria on the microbial structure and function of *Moringa* rhizosphere soil. This multi-dimensional study is helpful to understand the role of beneficial fungi and bacteria in the entire ecosystem of *M. oleifera*.
